# Early infant growth impairment in the setting of frequent exclusive breast feeding: considering therapeutic options

**DOI:** 10.1136/bmjgh-2022-010118

**Published:** 2022-08-29

**Authors:** Victoria Nankabirwa, Valerie J Flaherman, Raimundo Co, Amy Sarah Ginsburg, Augusto Braima de Sa

**Affiliations:** 1 School of Public Health, Makerere University, Kampala, Uganda; 2 Pediatrics, University of California San Francisco, San Francisco, California, USA; 3 International Partnership for Human Development, Bissau, Guinea-Bissau; 4 Clinical Trials Center, University of Washington, Seattle, Washington, USA

**Keywords:** Randomised control trial, Paediatrics, Nutrition, Child health, Epidemiology

Early, frequent exclusive breast feeding (EBF) has many important health benefits for infants through 6 months of age. Overall, EBF promotes optimal infant growth, critically important in low-income and middle-income countries (LMIC) where more than a third of young children suffer from growth impairment causing high risk of morbidity and mortality.^1^ Such growth deficits often begin during the first 6 months of life,^2^ and thus, the benefits of EBF can have a large population impact.^3^

Best practices for EBF are vitally important and should be a cornerstone of public health programs. Unfortunately, even in settings of best practices for EBF, early infant growth impairment can occur. For example, in separate randomised controlled trials in Guinea-Bissau and in Uganda, Burkina Faso and South Africa, implementation of best practices to support EBF did not improve infant growth or reduce the prevalence of early impairment in Guinea-Bissau or Uganda.^4 5^ Since best practices to support EBF did not reduce the prevalence of infant growth impairment in these locations, additional strategies are being investigated.

To identify a strategy that has the potential to reduce the prevalence of infant growth impairment in locations where best practices to support EBF did not, our study team conducted initial stakeholder visits and qualitative data collection in Guinea-Bissau, Nepal, Pakistan and Uganda, including focus groups and key informant interviews to explore local attitudes and experiences related to newborn feeding. Both in Guinea-Bissau and Uganda, there was a high degree of local concern regarding failure to thrive among some EBF infants and high interest in developing therapeutic options. Observational data in these locations showed that the risk of being underweight at 30 days of age was increased 6-fold among infants who were low birth weight and 10-fold among those who weighed<2600 g on the 4th day after birth.^6^

Based on this qualitative and observational data as well as on stakeholder feedback, the Preventing Infant Malnutrition with Early Supplementation (PRIMES) pilot randomised controlled trial was designed to test the efficacy of supplementing early breast feeding with a small daily volume of formula to improve growth for breastfeeding infants identified as at-risk due to risk factors demonstrated by the initial observational work.^7^ The trial’s initial design was modified based on feedback from ethical committees overseeing the study, including the Makerere University School of Public Health Research and Ethics Committee, the Uganda National Council of Science and Technology, the Guinea-Bissau Committee of Health and Ethics and the Institutional Review Board of the University of California San Francisco. PRIMES has been monitored by a Data Safety and Monitoring Board consisting of physicians, scientists and epidemiologists from Africa, Europe and the USA.

A recent commentary critical of PRIMES argued that studying any supplementation of breast feeding with formula is unethical.^8^ Respectfully, we disagree with this opinion and believe that studying supplementation of breast feeding with formula as a therapeutic option is ethically necessary when equipoise exists regarding a specific population’s growth not being optimally supported by EBF. We strongly advocate for the opportunity for academic discourse.

However, of concern, this same commentary implied that the regulatory and ethical approvals granted in Guinea-Bissau and Uganda were influenced by the availability of research funds for study activities. This implication is particularly distressing because all regulatory authorities and ethical committees approving and monitoring PRIMES have been highly engaged and active in evaluating the study and modifying its design to ensure appropriateness for local context. We find problematic the commentary authors’ statements denigrating these oversight groups for their differing perspectives and scientific opinions. Local conditions may and should influence regulatory and ethical bodies in LMIC.

Equity is fundamental to ethical global health research, and our team will continue to strive for this goal through local stakeholder engagement, rigorous data collection, compliance with all regulatory and ethical requirements, and evidence-based evaluation, while ensuring scientific merit, integrity and respect for those we serve. We welcome criticism, suggestions for improvement and alternative ideas. At the same time, unnecessary polarisation runs the risk of repressing legitimate scientific inquiry, undermining progress and contributing to the ever-increasing inequity between those who are able to achieve adequate nutrition and those who cannot. In the end, vulnerable mothers and their at-risk infants suffer. We hope all who care for small and at-risk infants can join together to work to improve infant nutrition, growth and health worldwide.

## Data Availability

There are no data in this work.
